# Editorial: The gut-skin axis: interaction of gut microbiome and skin diseases

**DOI:** 10.3389/fmicb.2024.1427770

**Published:** 2024-06-04

**Authors:** Jianmin Chai, Feilong Deng, Ying Li, Xiaoyuan Wei, Jiangchao Zhao

**Affiliations:** ^1^Guangdong Provincial Key Laboratory of Animal Molecular Design and Precise Breeding, College of Life Science and Engineering, Foshan University, Foshan, China; ^2^Division of Agriculture, Department of Animal Science, University of Arkansas, Fayetteville, AR, United States; ^3^Department of Food Science, The Pennsylvania State University, University Park, PA, United States

**Keywords:** gut-skin axis, microbiome, skin disease, skin epidermis, gut microbiome, microbiome-dynamics

More and more evidence has demonstrated that gut microbiome plays critical roles in modulating the development of diseases beyond the gastrointestinal tract, including skin disorders such as psoriasis. The gastrointestinal (GI) tract is one of the largest interfaces between the host and its environment, and is colonized by a large number of microbes, which have a big impact on host health (Deng et al., 2023; Chai et al., 2024). The skin epidermis, with appendage structures, such as sweat and sebaceous glands, have a total skin surface of about 25 m^2^ and is one of the largest epithelial surfaces for interaction with microbes. Moreover, both gut and skin act as barriers for immune function. Recent multi-omics technological advances have revealed the importance of the microbiome in health and diseases. Thus, deep investigation of the roles of the microbiome in skin disease will contribute to unveil the mechanisms of the gut-skin axis. In addition, it is necessary to better characterize the skin microbiota and its modes of interaction with the host's immune system.

The new conception “gut-skin axis” refers to the bidirectional relationship between the gut microbiome and skin health. Several mechanisms, such as intestinal barrier, inflammatory mediators, and metabolites, have been proposed for gut-skin axis. Currently, there have been various studies regarding the presence of the gut-skin axis and its resulting inflammatory effect due to gut microbiome imbalance. In addition, dysbiosis of the skin and gut microbiota is also observed in skin disorders. Therefore, understanding the gut-skin axis, especially in terms of microbiome modulation, is important for the gut and skin health, which may lead to development of novel therapies for skin disease.

A total of 11 articles published in this Research Topic broaden our knowledge of microbiome and common skin diseases. Psoriasis, a common erythematous scaling skin disease with multiple skin manifestations and systemic involvement, can involve any skin site and occur at any age and in any geographic area, affecting more than 60 million adults and children worldwide. Zhu et al. summarized the latest information on the unique patterns of gut microbiota and co-metabolites involved in the pathogenesis of psoriasis and attempt to explore microbial-based therapeutic targets derived from mono-and polymicrobial probiotics, fecal microbiota transplantation, pharmacomicrobiomics, and dietary interventions as diagnostic or therapeutic approaches promising to provide new options and long-term management for psoriasis. Another skin disease, alopecia areata, is a type of autoimmune inflammatory dermatological disease characterized by non-scarring hair loss of the scalp or body skin. Liu and Liu highlighted the relationship between alopecia areata and the gut microbiome or metabolome to provide novel directions for the prevention, clinical diagnosis and treatment of alopecia areata. Sánchez-Pellicer et al. reviewed the pathogenesis of rosacea in terms of gut-skin axis. Rosacea is a chronic skin disease affecting ~5.5% of the general population, mainly patients between 45 and 60 years old, which can severely impact the patient's quality of life as well as their mental health. Oral probiotic supplementation may be an alternative approach to improve the clinical evolution of rosacea. Systemic lupus erythematosus is a chronic autoimmune disease. Zhao et al. conducted a comprehensive analysis of 218 research articles and 118 review articles, and found that China and the United States have emerged as the most active contributors in this domain. They also concluded that recent research endeavors have predominantly focused on exploring the phenomenon of microbiota dysbiosis, specifically pertaining to gut microbiota dysbiosis, alongside elucidating the intricate mechanisms and applications associated with SLE, thereby establishing it as a prominent area of investigation.

In addition, four researches also revealed the link between gut microbiotas and skin diseases. Wu et al. found independent causal relationships between four gut microbes and acne vulgaris, and revealed a genetic association between acne vulgaris patients and gut microbiota. Modulation of these four gut microbes might be an effective way to prevent and treat acne vulgaris. The chronic inflammatory skin disease Hidradenitis suppurativa (HS) is strongly associated with Crohn's Disease (CD). However, gut microbiota composition and diet interaction have not been compared in HS and CD. Cronin et al. compared the fecal microbiota and habitual diet of previously reported subjects with HS, patients with CD and healthy controls, which reported that the fecal microbiota may help identify patients with HS who are at greater risk for development of CD. Xue et al. conducted a bidirectional Mendelian randomization (MR) which found a potential causative link between gut microbiota and hypertrophic scarring, opening up new ways for future mechanistic research and the exploration of nanobiotechnology therapies for skin disorders. Song et al. confirmed the gut microbiome and its metabolites could maintain the intestine barrier homeostasis to resist the intestinal damage caused by the skin ulceration syndrome. Whether alterations in the gut microbiota are a consequence or cause of skin diseases remains to be determined. However, it is clear that gut microbiota plays a critical role in progress or prevention of skin diseases.

Beyond gut microbiota, the microbiota inhabiting the host skin is also important for the skin barrier. Seo et al. identified that *Cutibacterium, Corynebacterium, Staphyloccocus, unclassified Neisseriaceae*, and *Streptococcus* were major skin microbial taxa at the genus level, and fluctuated with the seasons. Moreover, functional profiling using KEGG provided clues on the impact of *Cutibacterium* on the host skin barrier. This study enhances our understanding of the skin microbiome and its interplay with skin characteristics. Li et al. classified the microbiome and lipid profiles on forearm and cheek of humans, which provides insights on the potential correlation between skin microbiome and lipids. Another study conducted by Zhang et al. isolated potential skin probiotics, *Pantoea eucrina KBFS172*, and verified the positive effects of this strain on repairing UV damage. All these findings provide the insights that microbiota is associated with skin physiological characteristics and disease.

The present Research Topic highlights the tight links between the gut or skin microbiota and various skin diseases. It also reveals the contributions of microbial metabolites to skin disease. These recent advancements in the field of microbiome in skin disease provide insights into how to manipulate either gut or skin microbiota to improve host skin health in future studies. Isolations of probiotics from the host might be a new and effective research direction.

## Author contributions

JC: Conceptualization, Data curation, Resources, Visualization, Writing – original draft. FD: Data curation, Resources, Writing – original draft. YL: Conceptualization, Funding acquisition, Project administration, Resources, Supervision, Writing – review & editing. XW: Conceptualization, Resources, Supervision, Writing – review & editing. JZ: Conceptualization, Funding acquisition, Project administration, Resources, Supervision, Visualization, Writing – review & editing.
